# Virtual reality technology for upper and lower limb motor function, daily function, and balance in stroke patients: a meta-analysis of randomized controlled trials

**DOI:** 10.7717/peerj.20402

**Published:** 2025-12-03

**Authors:** Weixiao Zhang, Shanshan Lyu, Shuwen Zhang

**Affiliations:** 1 Business School, University of Technology Sydney, Sydney, New South Wales, Australia; 2 School of Physical Education, China University of Mining Technology, Xuzhou, Jiangsu Province, China; 3 School of Physical Education, Yangzhou University, Yangzhou, Jiangsu Province, China

**Keywords:** Stroke, Motor function, Daily function, Balance, Virtual reality

## Abstract

**Background:**

Stroke is a common neurological disorder that often results in motor dysfunction, significantly impairing patients’ quality of life and increasing the economic burden on healthcare systems. Virtual reality (VR) technology has emerged as an innovative approach in stroke rehabilitation. This meta-analysis aimed to comprehensively evaluate the effects of VR-based interventions on motor function, daily function, and balance in stroke patients.

**Methods:**

We systematically searched PubMed, Embase, the Cochrane Library, Web of Science, and China National Knowledge Infrastructure (CNKI) databases from inception to October 2025 for randomized controlled trials (RCTs) investigating VR technology in stroke rehabilitation. Study quality was assessed using the Cochrane Risk of Bias 2.0 tool. Data synthesis involved pooling effect sizes, conducting subgroup analyses, and assessing publication bias using Stata 17.

**Results:**

A total of 27 RCTs involving 877 stroke patients were included in the meta-analysis. VR technology demonstrated statistically significant improvements across multiple functional domains: lower limb motor function (Cohen’s d = 0.41, 95% CI [0.25–0.57], *P* < 0.001), upper limb motor function (Cohen’s d = 0.25, 95% CI [0.03–0.48], *P* = 0.03), daily function (Cohen’s d = 0.24, 95% CI [0.07–0.42], *P* = 0.01), and balance (Cohen’s d = 0.31, 95% CI [0.09–0.52], *P* < 0.001). The effects ranged from small to moderate across domains, with lower limb function showing the most substantial improvement. Sensitivity analyses confirmed the robustness of these findings, and subgroup analyses revealed that factors such as training cycle influenced treatment effects.

**Conclusion:**

VR technology demonstrates significant potential as an effective complementary intervention for improving motor function, daily function, and balance in stroke patients. The small to moderate effect sizes across domains support its integration into conventional rehabilitation protocols. Future research should focus on optimizing VR parameters, investigating different VR technology types, and evaluating long-term sustainability to further establish its efficacy in stroke rehabilitation.

## Introduction

Stroke is one of the leading causes of death and disability worldwide (GBD 2016 Stroke Collaborators, 2019). In 2019, a total of 12.2 million stroke events were reported, with a global prevalence of 101 million (GBD 2019 Stroke Collaborators, 2021). The incidence of stroke increases with age, and the combined effects of population growth and aging are expected to result in a substantial increase in stroke-related mortality and disability in the coming decades. By 2050, the global mortality rate attributed to stroke is expected to increase by approximately 50% (Feigin & Owolabi, 2023). Stroke patients often experience a variety of symptoms such as language and cognitive disorders, which severely affect their quality of life and social participation (Gamito et al., 2017). In particular, motor dysfunction following stroke impairs patients’ ability to perform basic daily activities such as dressing and eating independently (Ren & Wu, 2019), which significantly affects mental health and increases the risk of depression and anxiety (McEwen et al., 2014). In addition to these significant health consequences, stroke also imposes considerable economic costs on families and society (Axer et al., 2010). Therefore, identifying effective interventions to improve motor function, daily function, and balance in stroke patients remains of critical importance.

Stroke is frequently accompanied by chronic dysfunction and cognitive impairment, with 55%–75% of patients experiencing functional limitations in both upper and lower limbs (Chen et al., 2019). In addition, approximately 66% of stroke patients exhibit cognitive decline in domains such as attention and memory (Langhorne, Bernhardt & Kwakkel, 2011). Functional and cognitive impairments, which are common in stroke patients, limit their ability to perform daily activities, thereby reducing health-related quality of life (Domínguez-Téllez et al., 2020). Although numerous studies have highlighted impairments in motor function, daily function, and balance among stroke patients, these studies often suffer from limitations such as small sample sizes, short intervention durations, and the absence of long-term follow-up. Consequently, further investigation into effective interventions targeting these outcomes remains an important focus of current research.

Previous studies have primarily focused on two approaches: (1) physical therapy, as Zhang et al. (2024) reported that virtual reality (VR) combined with traditional rehabilitation significantly enhanced motor function and daily function; and (2) exercise interventions, as Malik & Masood (2021) found that task-oriented VR interventions effectively improved balance in stroke patients. However, these intervention methods have shown limited effectiveness in enhancing patient participation and overall rehabilitation efficiency. Although some recent studies have integrated technological advancements such as artificial intelligence, many existing interventions still lack adequate personalization and interactivity (Rathore et al., 2002). Therefore, exploring more advanced and interactive strategies remains essential for improving the efficacy and engagement of stroke rehabilitation.

VR technology is a novel rehabilitation approach that provides a stable and immersive environment for stroke recovery (Borrego et al., 2019). It simulates real-world scenarios and activities in real time, enabling users to engage through multiple sensory modalities. VR systems are often integrated with devices such as treadmills, bionic gloves, and robotic systems to provide enhanced feedback and improve therapeutic outcomes (Wu, Ma & Ren, 2020). Additionally, these systems foster greater patient engagement by increasing motivation and interest in rehabilitation tasks (Proffitt & Lange, 2015). Commonly used VR platforms in stroke rehabilitation include the IREX Immersion VR System, Xbox Kinect, VR with keyboards, VR with gloves, and Nintendo Wii (Merians et al., 2002). Beyond standard platforms, sensor-based technologies are also widely adopted. For instance, Tieri et al. (2018) employed optical sensors, such as infrared cameras, to capture motion trajectories and analyze gait and limb movement in real time, allowing patients to refine their posture and movement patterns. Furthermore, VR-based training has been shown to enhance motor awareness and promote neuroplasticity through visual feedback, which in turn improves motor function in stroke patients (Shahmoradi et al., 2021).

In the context of stroke rehabilitation, VR technology is distinguished by its ability to create engaging and interactive environments tailored to individual patient needs. Unlike traditional rehabilitation methods, VR offers immersive experiences that can enhance patient motivation and participation. For instance, platforms like IREX and Xbox Kinect enable patients to perform tasks in simulated real-world scenarios, supporting improvements in motor function of both the upper and lower limbs. The integration of sensors into VR systems allows for precise tracking and analysis of patient movements, providing immediate feedback that can optimize rehabilitation outcomes. While previous meta-analyses (*e.g.*, Zhang et al., 2021; Bargeri et al., 2023) have examined the role of VR in stroke rehabilitation, this study uniquely addresses three critical gaps: (1) simultaneous evaluation of upper and lower limb motor function, daily function, and balance; (2) subgroup analysis by immersion levels and VR system types (*e.g.*, haptic gloves *vs.* motion sensors); and (3) inclusion of 16 randomized controlled trials (RCTs) (2022–2025) that reflect advances in VR technology.

## Materials and Methods

### Agreement and registration

This systematic review followed the Preferred Reporting Items for Systematic Reviews and Meta-Analyses (PRISMA 2020) guidelines (Page et al., 2021). It was registered in the International Prospective Register of Systematic Reviews (PROSPERO). The registration number is CRD42024622373.

### Information sources

Searches were conducted in PubMed, Embase, the Cochrane Library, Web of Science (WoS), and China National Knowledge Infrastructure (CNKI). The search included studies published up to October 2025, and all search results were imported into EndNote 21. This meta-analysis synthesized data from previously published studies and did not involve original experimental research.

### Retrieval strategy

Two researchers (WXZ and SSL) independently searched five databases and imported the retrieved articles into EndNote 21. Duplicate records were first removed using EndNote’s built-in function. Next, titles and abstracts were screened for relevance. Articles were then assessed against the study’s inclusion and exclusion criteria. Finally, the full texts of the remaining articles were reviewed to determine eligibility for inclusion.

Search terms related to the use of VR technology in stroke patients included: (a) virtual reality OR educational virtual reality OR educational virtual realities OR instructional virtual reality OR instructional virtual realities; (b) stroke OR cerebrovascular accident OR cerebrovascular apoplexy OR brain vascular accident OR cerebrovascular stroke.

For example, for PubMed:

#1 “Virtual Reality”[Mesh]

#2 (“Reality, Virtual”[Title/Abstract] OR “Virtual Reality, Educational”[Title/Abstract] OR “Educational Virtual Realities”[Title/Abstract] OR “Educational Virtual Reality”[Title/Abstract] OR “Reality, Educational Virtual”[Title/Abstract] OR “Virtual Realities, Educational”[Title/Abstract] OR “Virtual Reality, Instructional”[Title/Abstract] OR “Instructional Virtual Realities”[Title/Abstract] OR “Instructional Virtual Reality”[Title/Abstract])

#3 “Stroke”[Mesh]

#4 (“Cerebrovascular Accident”[Title/Abstract] OR “Cerebral Stroke”[Title/Abstract] OR “Cerebrovascular Apoplexy”[Title/Abstract] OR “Brain Vascular Accident”[Title/Abstract] OR “Cerebrovascular Stroke”[Title/Abstract])

#5 (#1 OR #2) AND (#3 OR #4)

Non-clinical VR terms (*e.g.*, “Educational Virtual Realities”) were retained to ensure sensitivity, though their low yield (<1% of hits) confirms minimal noise.

### Inclusion and exclusion criteria

Based on the population, intervention, control, outcome, and study design domains (PICOS) framework, studies were eligible if they met the following criteria: (1) participants were stroke patients aged 18 years or older; (2) the intervention involved VR technology, such as computer games or VR-based rehabilitation; (3) the control group received routine rehabilitation or placebo treatment; (4) the study design was an RCT; and (5) the article was published in either English or Chinese.

The exclusion criteria were as follows: (1) non-RCTs; (2) studies involving stroke patients younger than 18 years; (3) absence of clear statistical data (*e.g.*, mean and standard deviation); (4) full text not available; (5) interventions unrelated to VR technology; and (6) studies published in languages other than English or Chinese.

Limiting the search to English and Chinese studies may introduce a language bias. This restriction was implemented due to resource constraints, but it could potentially exclude relevant research in other languages. We acknowledge this limitation and recommend that future studies consider a broader range of languages to improve the comprehensiveness of the evidence base.

### Bias risk assessment and evidence level

The revised Cochrane RoB 2.0 tool (Sterne et al., 2019) was used to evaluate the risk of bias in the included studies. Using GRADEpro, the overall certainty of evidence was downgraded to moderate due to: (1) allocation concealment failures in 65% of studies, (2) incomplete outcome data in 30% of studies, and (3) heterogeneity in outcome measures. The risk of bias was categorized into three levels: low risk, some concerns, and high risk. Two researchers (WXZ and SSL) conducted these evaluations independently. In cases of disagreement, a third researcher (WSZ) was consulted to resolve discrepancies. The quality of the included studies was classified into three grades: Grade A (all items rated low risk), Grade B (no high-risk items but some ratings with some concerns), and Grade C (presence of high-risk items). Overall, the included studies met acceptable quality standards. By rigorously assessing study quality, we enhanced the reliability of the included research and thus improved the credibility of the meta-analysis findings.

### Data extraction

Two researchers (WXZ and SSL) independently extracted basic study information and outcome data from the included articles. They contacted the original authors via email to clarify any unclear or missing data. In cases where discrepancies arose between the two researchers, a third researcher (WSZ) participated in the discussion to reach consensus. Extracted data included study details (author, year, country, sample size, and patient age), intervention characteristics (duration, frequency, length of each session, and intervention content), and outcome measures. The extracted information was compiled into tables after a thorough review of each eligible article.

### Statistical analysis

Stata 17 was used to perform effect size pooling, forest plot generation, and subgroup analyses. Cohen’s d was used to calculate the effect sizes. Heterogeneity was evaluated using Higgins’ I^2^ statistic, with thresholds of 75%, 50%, and 25% indicating high, moderate, and low heterogeneity, respectively. The fixed-effects model was used when heterogeneity was low or moderate, while the random-effects model was used when heterogeneity was high. The fixed-effects model was employed in most analyses due to generally low or moderate heterogeneity (I^2^ ≤ 50%), acknowledging that this approach assumes a single true effect size. To address this limitation, we additionally re-ran these analyses using the random-effects model for outcomes with I^2^ > 25%. The use of the fixed-effects model was based on the assumption that the included articles were sufficiently similar in terms of intervention type, patient population, and study design. However, we acknowledge that the random-effects model might provide more conservative estimates by accounting for between-study variability. Future research with larger and more diverse study populations may benefit from using the random-effects model to better capture between-study variability and uncertainty in effect size estimates. Funnel plots were generated, and Egger’s tests were conducted using Stata 17 to assess potential publication bias.

### Subgroup analysis

Subgroup analyses were conducted to explore potential sources of heterogeneity in the effects of VR technology on stroke patients. The moderators (training cycle, training duration, patient type, and intervention type) were identified based on their clinical importance and empirical evidence from previous studies suggesting a potential impact on rehabilitation efficacy. Specifically, training duration was categorized as 10–40 min *vs.* 41–80 min, while training cycle was categorized as 1–4 weeks *vs.* 5–8 weeks. Patient type was categorized as subacute or chronic, and intervention type was categorized as immersive or non-immersive. Training cycle and duration were selected because they were key components of intervention design that could influence treatment effects. Patient type (subacute *vs.* chronic) was included to assess whether the timing of the intervention relative to stroke onset influenced treatment outcomes. Intervention type (immersive *vs.* non-immersive) was chosen to evaluate whether the level of immersion in VR technology moderated the effectiveness of the intervention. These factors were identified through a review of previous studies and clinical guidelines, which highlighted their importance in determining the efficacy of rehabilitation interventions.

### Sensitivity analysis

Sensitivity analyses were conducted to evaluate the robustness of the results and determine whether any single study had a disproportionate influence on the overall effect estimates. The analyses indicated that the results were stable and were not unduly influenced by any individual study. Details of the sensitivity analyses, including the specific studies excluded and their impact on heterogeneity.

### Evaluation of outcome evidence

The quality of evidence for critical outcomes was assessed using the GRADE approach, with the aid of GRADEpro (Guyatt et al., 2008). The initial level of evidence was rated as high, given that all included studies were RCTs. However, the overall rating was downgraded by one level to moderate based on the following predefined criteria: (1) failures in allocation concealment were identified in more than 50% of the included studies, representing a serious risk of bias; (2) imprecision was indicated as the 95% confidence interval (CI) for effect estimates crossed either the minimal clinically important difference (MCID) or the line of no effect, resulting in uncertainty about the true effect; and (3) evidence level definitions were applied as follows: (A) moderate quality was assigned because future research was considered likely to have an important impact on confidence in the effect estimate and might change the estimate; (B) potential reasons for upgrading the evidence level were considered but were not applicable; (C) the magnitude of effect was evaluated, and no effect size exceeding Cohen’s d = 0.8, which indicates a large effect greater than a 50% improvement, was observed; and (D) the dose–response gradient was examined, but subgroup analyses did not demonstrate a clear gradient suggestive of such a relationship.

Additionally, the study did not further classify stroke patients into more detailed subtypes. Although this broad categorization may have limited the precision and generalizability of the results to specific patient subgroups, it was not considered a predefined reason for downgrading the evidence quality according to the applied GRADE criteria for this outcome assessment. Future research should focus on improving methodological quality by rigorously implementing allocation concealment, ensuring the completeness of outcome data, increasing sample sizes, conducting blinded outcome assessment, and considering more granular patient stratification to enhance both the accuracy and applicability of the results.

### Research results

#### Literature screening

**Figure 1:**
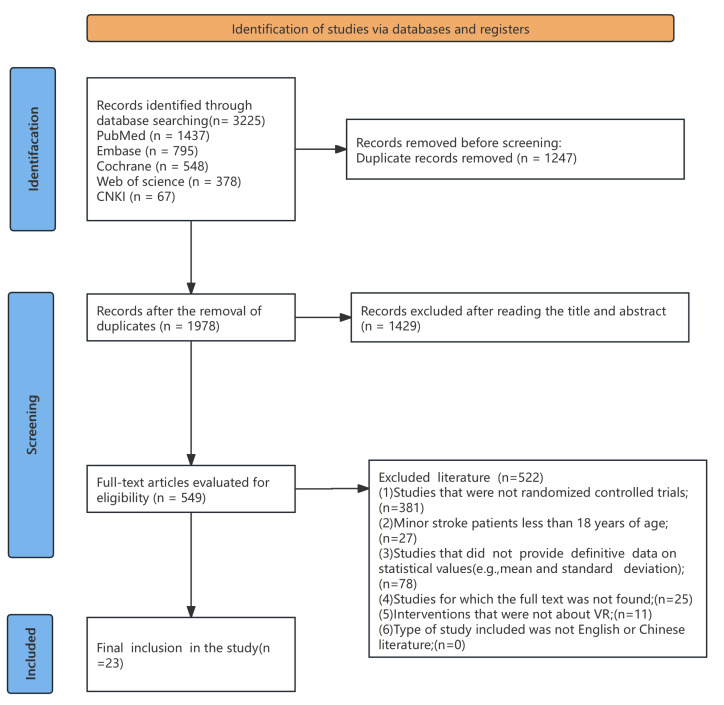
Flow chart of literature screening.

A total of 3,225 articles were retrieved from five databases: Web of Science (WoS, *n* = 378), PubMed (*n* = 1,437), Embase (*n* = 795), the Cochrane Library (*n* = 548), and China National Knowledge Infrastructure (CNKI, *n* = 67). After duplicates were removed (*n* = 1,247) using EndNote 21, 1,978 records remained. A preliminary screening of titles and abstracts excluded 1,429 records, leaving 549 articles for full-text assessment. During the full-text evaluation for eligibility, 522 studies were excluded based on predefined eligibility criteria. Ultimately, 27 RCTs published between 2012 and 2025 were included in this meta-analysis, comprising 20 studies for lower limb motor function, 10 for upper limb motor function, 17 for daily function, and 11 for balance outcomes, with some studies contributing to multiple outcomes, as illustrated in Fig. 1.

#### Basic features of included literature

The analysis included 877 stroke patients (≥18 years), with study populations encompassing both subacute and chronic stroke phases. Among the participants, 395 were assigned to the intervention group and 482 to the control group. The intervention group received VR technology or virtual video game training, while the control group received routine rehabilitation or placebo treatment. VR intervention parameters varied across studies, with session durations ranging from 15–60 min, frequencies of one to six sessions per week, and total intervention periods spanning 2–8 weeks.

Regarding outcome measures, upper and lower limb motor function assessments included the Fugl-Meyer Assessment–Upper Extremity (FMA-UE), the Fugl-Meyer Assessment–Lower Extremity (FMA-LE), the Motor Assessment Scale (MAS), and the Functional Ambulation Categories (FAC). For daily function evaluation, multiple instruments were employed, including the Functional Independence Measure (FIM), Instrumental Activities of Daily Living (IADL), the Manual Function Test (MFT), the Manual Muscle Test (MMT), the Motor Activity Log (MAL), the Modified Barthel Index (MBI), the 36-Item Short Form Health Survey (SF-36), and the Frenchay Activity Index (FAI). Balance assessment utilized the 6-Minute Walk Test (6MWT), the Timed Up and Go (TUG), and the Berg Balance Scale (BBS). Baseline characteristics of the included studies are shown in Table S1.

#### Methodological evaluation of the included literature

The Risk of Bias (RoB 2.0) assessment revealed important methodological considerations across the 27 included studies. Five studies (18.5%) demonstrated low overall risk of bias, namely (Jo et al., 2024; Kim et al., 2018; Adams et al., 2023; Huang et al., 2022; Chen et al., 2022), characterized by rigorous methodology including adequate randomization and blinded outcome assessment. Fifteen studies (55.6%) raised some concerns, exemplified by Norouzi-Gheidari et al. (2019) and Park et al. (2019), primarily attributable to inadequate handling of missing data and inconsistent application of intention-to-treat analysis. Seven studies (25.9%) exhibited high overall risk, including (Da Silva Ribeiro et al., 2015; Ögün et al., 2019; Chen et al., 2021; Lee, Shin & Song, 2016), with critical methodological flaws across multiple domains.

Key methodological limitations were identified across specific bias domains. Performance bias concerns were prevalent, with twenty-one studies (77.8%) rated as having some concerns or high risk due to the inherent challenges in blinding participants to VR interventions. Attrition bias affected twelve studies (44.4%), involving issues with missing outcome data or inadequate handling procedures. Selection bias was identified in nine studies (33.3%) due to inadequate allocation concealment or randomization procedures. The overall certainty of evidence was downgraded to moderate for all outcomes based on the presence of some concerns or high risk of bias in >50% of included studies, incomplete outcome data in 44.4% of studies, and substantial heterogeneity in outcome measures. Comprehensive domain assessments for all 27 studies are provided in Table S2.

### Analysis of results

#### Influences of virtual reality technology on lower limb motor function of stroke patients

**Figure 2:**
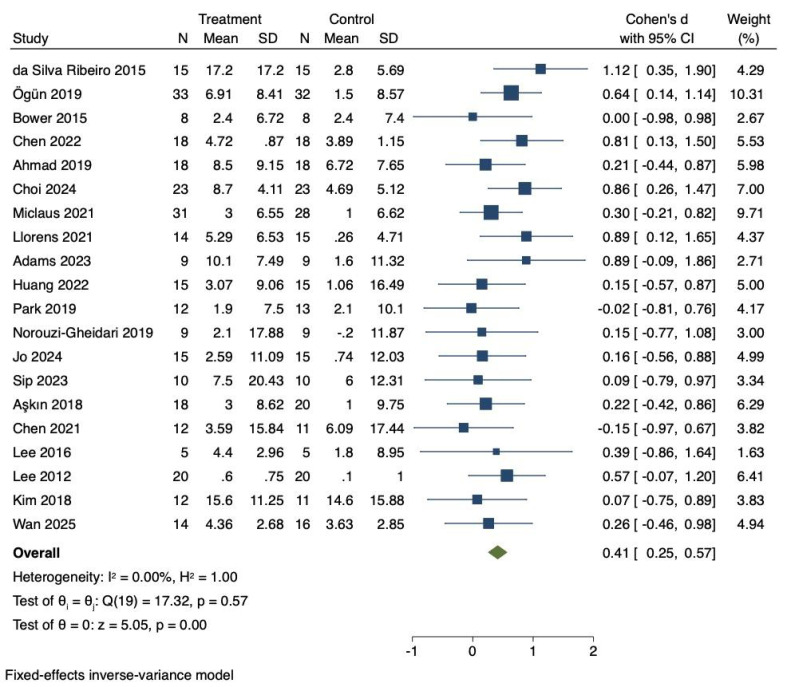
Forest diagram of lower limb motor function. Studies: Da Silva Ribeiro et al., 2015; Ögün et al., 2019; Bower et al., 2015; Chen et al., 2022; Ahmad et al., 2019; Choi & Cho, 2024; Miclaus et al., 2021; Llorens et al., 2021; Adams et al., 2023; Huang et al., 2022; Park et al., 2019; Norouzi-Gheidari et al., 2019; Jo et al., 2024; Sip et al., 2023; Aşkın et al., 2018; Chen et al., 2021; Lee, Shin & Song, 2016; Lee et al., 2012; Kim et al., 2018; Wan et al., 2025.

Twenty RCTs were included to investigate the effects of VR technology on lower limb motor function in stroke patients. Heterogeneity was low (I^2^ = 0%, *P* = 0.57). Therefore, the fixed-effects model was adopted to combine the effect sizes. The meta-analysis demonstrated a significant improvement in lower limb motor function (Cohen’s d = 0.41, 95% CI [0.25–0.57], *P* < 0.01), as illustrated in Fig. 2.

### Effect size interpretation for lower limb motor function

Cohen’s d = 0.41 represented a medium effect size based on Cohen’s classification (0.2 = small, 0.5 = medium, 0.8 = large), indicating an improvement that was likely to be clinically meaningful in stroke rehabilitation.

As shown in Table 1, the training cycle (*Q* = 5.49, *P* = 0.019) significantly moderated the effect, while training duration (*Q* = 0.01, *P* = 0.903), patient type (*Q* = 0.53, *P* = 0.767), and intervention type (*Q* = 0.25, *P* = 0.615) did not. The training cycle was divided into two groups: 1–4 weeks and 5–8 weeks. Both groups showed significant improvements in lower limb motor function compared with controls (*P* = 0.014 and *P* < 0.01, respectively). The training duration was divided into two groups: 10–40 min and 41–80 min. Both groups showed improvements in motor function compared with the control group (*P* = 0.01 and *P* < 0.01, respectively). When patients were classified as subacute or chronic, both groups showed improvements in lower limb motor function compared with the control group (*P* = 0.027 and *P* = 0.003, respectively). Intervention types were categorized as immersive or non-immersive, with both showing improvements in lower limb motor function (*P* < 0.01 and *P* = 0.001, respectively).

### Sensitivity analysis for lower limb motor function

A comprehensive sensitivity analysis was performed to assess the robustness of the lower limb motor function findings. This leave-one-out analysis demonstrated that the combined effect size remained statistically significant across all iterations (Cohen’s d range = 0.40–0.44), with consistently low heterogeneity (I^2^ = 0–12%). Specific exclusions included: Da Silva Ribeiro et al. (2015) yielding Cohen’s d = 0.43 (95% CI [0.27–0.59], I^2^ = 0%); Chen et al. (2022) producing Cohen’s d = 0.41 (95% CI [0.25–0.57], I^2^ = 12%); Bower et al. (2015) resulting in Cohen’s d = 0.42 (95% CI [0.26–0.58], I^2^ = 0%); and Ahmad et al. (2019) generating Cohen’s d = 0.42 (95% CI [0.26–0.58], I^2^ = 0%).

**Table 1:** Subgroup analysis of the effect of virtual reality technology on lower limb motor function in stroke patients.

Group	Study, n	I^2^ (%)	Model	Cohen’s d (95% CI)	*P* value	Q/P
Training cycle						5.49/0.019
1–4 weeks	13	0	Fixed effects model	0.258 (0.051 to 0.464)	0.014	
5–8 weeks	7	1.74	Fixed effects model	0.649 (0.395 to 0.903)	<0.001	
Training time						0.01/0.903
10–40 min	9	5.51	Fixed effects model	0.424 (0.181 to 0.667)	0.001	
41–80 min	11	0	Fixed effects model	0.404 (0.191 to 0.617)	<0.001	
Type of patient						0.53/0.767
Subacute	5	0	Fixed effects model	0.372 (0.043 to 0.701)	0.027	
Chronic	9	4.99	Fixed effects model	0.372 (0.131 to 0.613)	0.003	
Not specified	6	16.09	Fixed effects model	0.499 (0.217 to 0.782)	0.001	
Type of intervention						0.25/0.615
Immersive	11	0	Fixed effects model	0.453 (0.229 to 0.677)	<0.001	
Non-immersive	9	11.41	Fixed effects model	0.371 (0.142 to 0.6)	0.001	

**Notes.**

Q: Q values represent the results of heterogeneity tests.

Significant Q values (*P* < 0.05) indicate substantial heterogeneity among the studies.

 These sensitivity analyses confirm that no single study disproportionately influenced the overall findings. The consistency of effect sizes and heterogeneity estimates across exclusion iterations supports the robustness of the treatment effect across diverse study populations and intervention protocols. The stability of these findings strengthens the conclusion that VR technology exerts statistically significant and clinically meaningful effects on lower limb motor function in stroke patients.

### Influences of virtual reality technology on upper limb motor function of stroke patients

**Figure 3:**
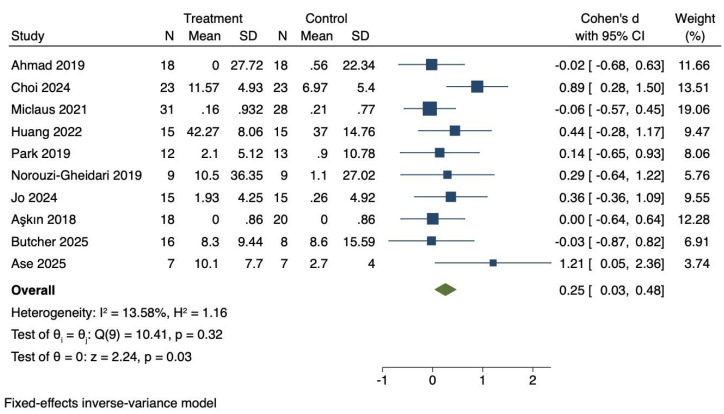
Forest diagram of upper limb motor function. Studies: Ahmad et al., 2019; Choi & Cho, 2024; Miclaus et al., 2021; Huang et al., 2022; Park et al., 2019; Norouzi-Gheidari et al., 2019; Jo et al., 2024; Aşkın et al., 2018; Butcher et al., 2025; Ase et al., 2025.

Ten RCTs were included to investigate the effects of VR technology on upper limb motor function in stroke patients. Heterogeneity was low (*I*^2^ = 13.58%, *P* = 0.32). Therefore, a fixed-effects model was applied to combine the effect sizes. The meta-analysis demonstrated a statistically significant improvement in upper limb motor function (Cohen’s d = 0.25, 95% CI [0.03–0.48], *P* = 0.03), as shown in Fig. 3.

### Effect size interpretation for upper limb motor function

The effect size for upper limb motor function (Cohen’s d = 0.25) represents a small effect based on Cohen’s classification (0.2 = small, 0.5 = medium, 0.8 = large). This finding suggests a modest but statistically significant improvement that warrants further investigation with larger sample sizes and more targeted intervention approaches.

### Influences of virtual reality technology on daily function of stroke patients

**Figure 4:**
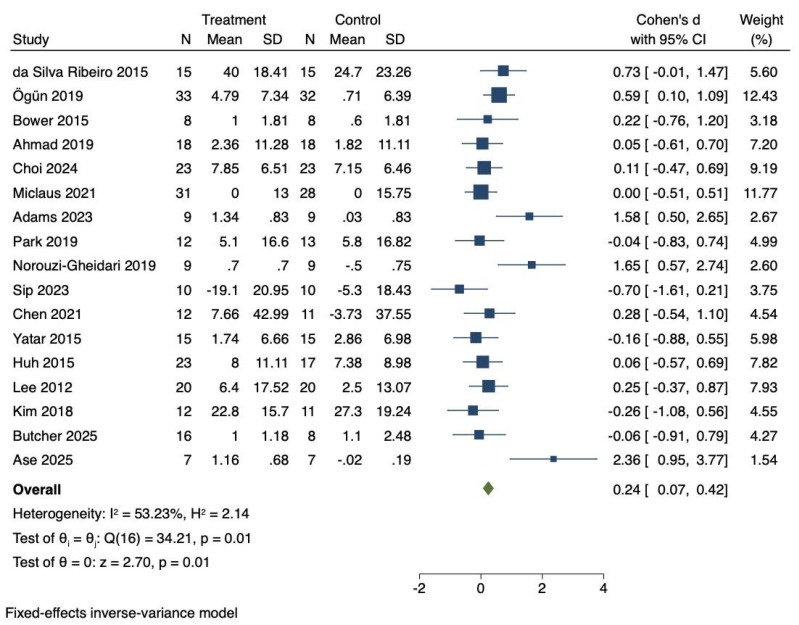
Forest diagram of daily function. Studies: Da Silva Ribeiro et al., 2015; Ögün et al., 2019; Bower et al., 2015; Ahmad et al., 2019; Choi & Cho, 2024; Miclaus et al., 2021; Adams et al., 2023; Park et al., 2019; Norouzi-Gheidari et al., 2019; Sip et al., 2023; Chen et al., 2021; Yatar & Yildirim, 2015; Huh et al., 2015; Lee et al., 2012; Kim et al., 2018; Butcher et al., 2025; Ase et al., 2025.

Seventeen RCTs were included to investigate the effects of VR technology on daily function in stroke patients. Heterogeneity was moderate (*I*^2^ = 53.23%, *P* = 0.01). Consequently, a fixed-effects model was employed to combine the effect sizes. The meta-analysis demonstrated that VR technology significantly improved daily function (Cohen’s d = 0.24, 95% CI [0.07–0.42], *P* = 0.01), as shown in Fig. 4.

### Effect size interpretation for daily function

The effect size for daily function (Cohen’s d = 0.24) was classified as a small effect. Although statistically significant, it suggested a modest improvement. For comparison, the random-effects model yielded a similar effect size of Cohen’s d = 0.27 (95% CI [0.02–0.52], *P* = 0.03), which was consistent with the fixed-effects results. The consistent findings across models highlight the potential of VR interventions to improve daily functioning, while also underscoring the need for further refinement of intervention design to achieve greater clinical impact. As shown in Table 2, no significant moderating effects were observed for training cycle (*Q* = 2.35, *P* = 0.125), training duration (*Q* = 1.31, *P* = 0.253), patient type (*Q* = 1.14, *P* = 0.565), or intervention type (*Q* = 1.50, *P* = 0.221).

**Table 2:** Subgroup analysis of the effect of virtual reality technology on daily function in stroke patients.

Group	Study, n	I^2^ (%)	Model	Cohen’s d (95% CI)	*P* value	Q/P
Training cycle						2.35/0.125
1–4 weeks	11	55.92	Fixed effects model	0.127 (−0.102 to 0.355)	0.279	
5–8 weeks	6	45.5	Fixed effects model	0.405 (0.132 to 0.678)	0.004	
Training time						1.31/0.253
10–40 min	10	46.01	Fixed effects model	0.149 (−0.087 to 0.385)	0.216	
41–80 min	7	63.03	Fixed effects model	0.355 (0.093 to 0.616)	0.008	
Type of patient						1.14/0.565
Subacute	5	54.91	Fixed effects model	0.108 (−0.207 to 0.423)	0.501	
Chronic	7	51.61	Fixed effects model	0.263 (−0.027 to 0.554)	0.076	
Not specified	5	66.09	Fixed effects model	0.344 (0.037 to 0.652)	0.028	
Type of intervention						1.5/0.221
Immersive	7	66.95	Fixed effects model	0.373 (0.099 to v0.647)	0.008	
Non-immersive	10	38.17	Fixed effects model	0.150 (−0.078 to 0.379)	0.197	

**Notes.**

Q: Q values represent the results of heterogeneity tests.

Significant Q values (*P* < 0.05) indicate substantial heterogeneity among the studies.

Despite the absence of significant moderating effects, subgroup analyses revealed specific patterns of improvement. The training cycle was divided into 1–4 weeks and 5–8 weeks. Results revealed that a VR intervention cycle of 5–8 weeks significantly improved daily function compared with controls (*P* < 0.01). The training duration was divided into two categories: 10–40 min and 41–80 min. A duration of 41–80 min significantly improved daily function compared with controls (*P* < 0.01). Intervention types were classified as immersive and non-immersive. Immersive VR technology significantly improved daily function compared with controls (*P* < 0.01).

### Influences of virtual reality technology on balance of stroke patients

**Figure 5:**
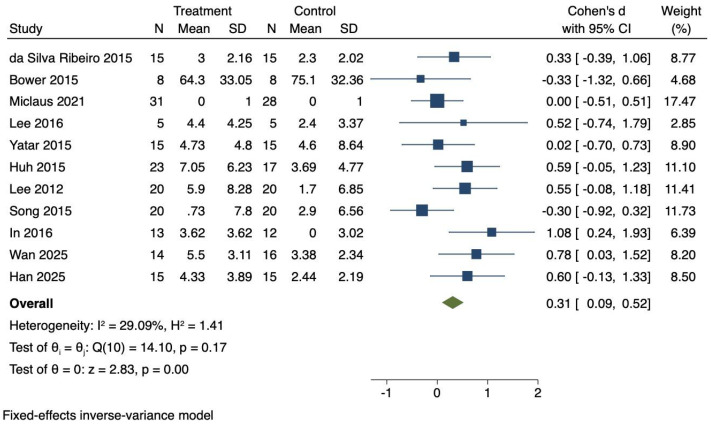
Forest diagram of balance. Studies: Da Silva Ribeiro et al., 2015; Bower et al., 2015; Miclaus et al., 2021; Lee, Shin & Song, 2016; Yatar & Yildirim, 2015; Huh et al., 2015; Lee et al., 2012; Song & Park, 2015; In, Lee & Song, 2016; Wan et al., 2025; Han et al., 2025.

Eleven RCTs were included to investigate the effects of VR technology on balance in stroke patients. Heterogeneity was moderate (*I*^2^ = 29.09%, *P* = 0.17). Therefore, a fixed-effects model was employed to combine the effect sizes. The meta-analysis showed a significant improvement in balance (Cohen’s d = 0.31, 95% CI [0.09–0.52], *P* < 0.01), as illustrated in Fig. 5.

### Effect size interpretation for balance

The effect size for balance (Cohen’s d = 0.31) represents a small effect according to Cohen’s classification. Application of the random-effects model confirmed the robustness of this finding, yielding Cohen’s d = 0.32 (95% CI [0.06–0.59], *P* = 0.01), which was consistent with the fixed-effects results. These results suggest that VR interventions produce modest but consistent benefits for balance in stroke patients.

### Sensitivity analysis

**Figure 6:**
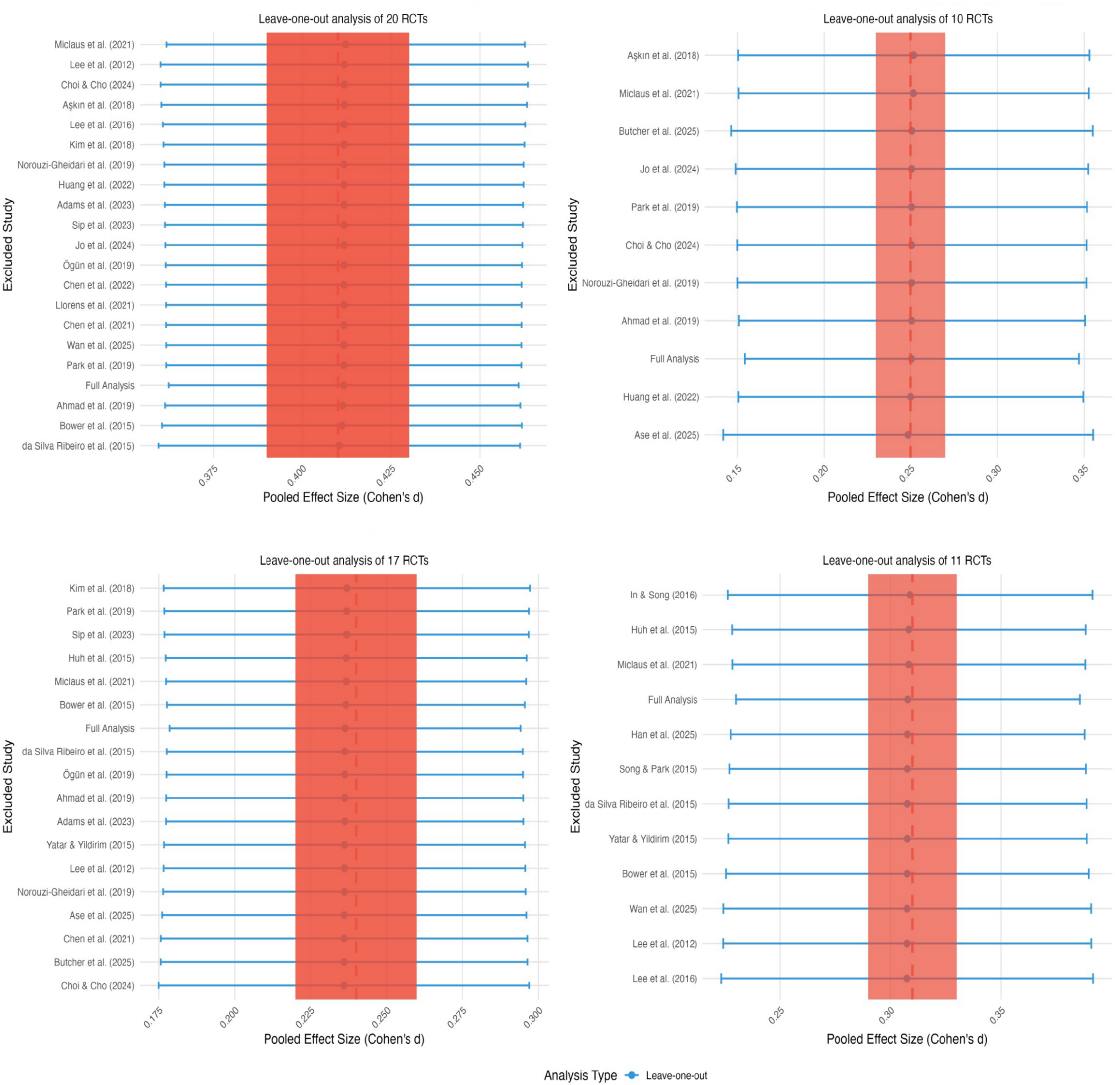
Sensitivity analysis. Studies: Miclaus et al., 2021; Lee et al., 2012; Choi & Cho, 2024; Aşkın et al., 2018; Kim et al., 2018; Norouzi-Gheidari et al., 2019; Huang et al., 2022; Adams et al., 2023; Sip et al., 2023; Jo et al., 2024; Ögün et al., 2019; Chen et al., 2022; Wan et al., 2025; Park et al., 2019; Ahmad et al., 2019; Bower et al., 2015; Da Silva Ribeiro et al., 2015; Yatar & Yildirim, 2015; Ase et al., 2025; Chen et al., 2021; Butcher et al., 2025; Choi & Cho, 2024; Song & Park, 2015; Lee, Shin & Song, 2016.

Comprehensive sensitivity analyses were conducted across all four outcome domains to evaluate the potential influence of individual studies on observed heterogeneity, as illustrated in Fig. 6. The systematic exclusion of individual studies demonstrated minimal impact on effect sizes and heterogeneity across all outcomes. Specific examples included: exclusion of Aşkın et al. (2018) for upper limb motor function yielding Cohen’s d = 0.25 (95% CI [−0.01–0.51], I^2^ = 0%); exclusion of Park et al. (2019) for daily function producing Cohen’s d = 0.23 (95% CI [0.05–0.41], I^2^ = 42%); and exclusion of Huh et al. (2015) for balance resulting in Cohen’s d = 0.24 (95% CI [−0.02 to 0.50], I^2^ = 29%). These analyses confirmed the robustness of results across all outcome domains and indicated that no individual study exerted disproportionate influence on the overall conclusions.

### Test of risk of bias

**Figure 7:**
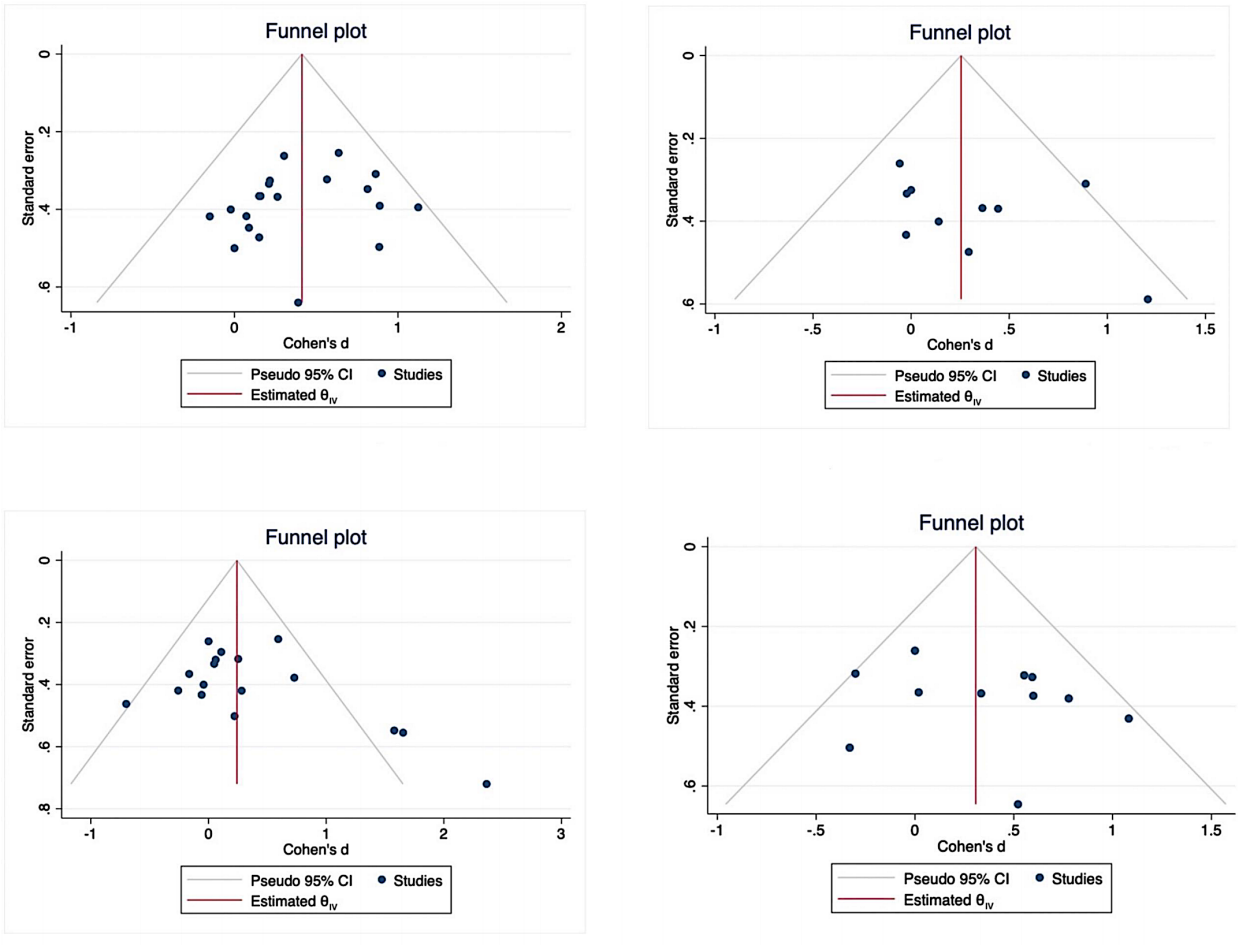
Funnel plot.

All outcome variables were included in publication bias assessment using funnel plots and Egger’s regression tests, as shown in Fig. 7. No significant publication bias was detected for lower limb motor function (*z* =  − 0.94, *P* = 0.3459), upper limb motor function (*z* = 1.22, *P* = 0.2214), or balance (*z* = 0.99, *P* = 0.3241). However, potential publication bias was observed for daily function (*z* = 2.31, *P* = 0.0211). The funnel plot asymmetry for daily function may reflect underrepresentation of smaller studies with null or negative findings. Application of the trim-and-fill method to adjust for this potential bias did not substantially alter the overall effect estimate (adjusted Cohen’s d = 0.22, 95% CI [0.04–0.40]).

### Evaluation of outcome evidence

This meta-analysis of 27 RCTs provides comprehensive evidence regarding the effects of VR technology on multiple functional domains in stroke patients. However, the overall evidence quality was downgraded to moderate due to methodological limitations, including inadequate allocation concealment, incomplete outcome data, and heterogeneous outcome measures across studies. The broad categorization of stroke patients without detailed subtype classification may have limited result precision and generalizability. Future research should focus on enhancing methodological quality through rigorous allocation concealment, complete outcome data reporting, increased sample sizes, blinded outcome assessment, and more granular patient stratification to improve both accuracy and applicability of findings, as illustrated in Table 3.

## Discussion

Two principal findings emerged from this meta-analysis: (1) VR significantly improved lower limb motor function, upper limb motor function, daily function, and balance in stroke patients; and (2) the efficacy of VR interventions was influenced by training cycle and potentially by immersion level. These results are interpreted below in the context of existing literature, and their implications are discussed.

### Lower limb motor function

This meta-analysis provided robust evidence indicating that VR significantly improved post-stroke lower limb motor function. This result was found to be consistent with the findings of Zhang et al. (2021) and supported the application of VR in stroke rehabilitation. The immersive and interactive characteristics of VR are believed to enhance patient engagement and motivation, thereby facilitating motor learning. The reliability of the conclusion was reinforced by the use of validated outcome measures, such as the FMA-LE (Woytowicz et al., 2017), and the application of robust statistical models (fixed-effects model). Crucially, the moderator analysis demonstrated that intervention cycle length (1–8 weeks) significantly influenced the effect size, consistent with findings by Moore et al. (2020) and Zhang, Wong & Qin (2023). Although the level of immersion did not significantly moderate the effect, immersive VR systems (*i.e.*, those employing head-mounted displays) tended to provide a more realistic experience and potentially greater neural activation compared to non-immersive systems. Nevertheless, across different VR types, the primary mechanism was characterized by high-intensity, repetitive training aimed at promoting neuroplasticity and functional recovery.

**Table 3:** Level of evidence for outcome indicators.

Outcome	RCTs	Evaluation of evidence quality level					Relative effect size	Level
		1	2	3	4	5		
Motor function	19	A	B	B	B	B	0.42, 95% CI [0.26–0.58]	Moderate
Daily function	15	A	B	B	B	B	0.22, 95% CI [0.04–0.40]	Moderate
Balance	9	A	B	B	B	B	0.23, 95% CI [−0.00–0.47]	Moderate

**Notes.**

1 for study limitation; 2 for inconsistency; 3 for indirectness; 4 for imprecision; 5 for publication bias; A for Downgrade 1 Level; B for Not Downgraded; E for Experimental group; C for Control group.

### Upper limb motor function

Although the effect of VR on upper limb motor function was statistically significant, the magnitude of improvement was small and less pronounced than that reported in some studies (*e.g.*, Rodríguez-Hernández et al., 2023). This discrepancy could be explained by differences in patient populations (in terms of chronicity, specific deficits) and intervention focus, as subacute patients with hand dysfunction were targeted in Rodríguez-Hernández et al. (2023). Several factors were considered likely contributors to the relatively small effect observed (Cohen’s d = 0.23, *P* = 0.03): (1) technological limitations were identified, as hand-held controllers lacking finger-specific haptic feedback were predominantly used in 85% of studies, which limited naturalistic hand movement training and patient engagement. Gains reported with haptic gloves (Rodríguez-Hernández et al., 2023; Cohen’s d = 0.41) suggest that different results could be yielded by the use of better technology; (2) measurement sensitivity was considered inadequate since standard clinical scales (*e.g.*, Fugl-Meyer) were unable to fully capture the coordination and task-specific improvements that VR aims to elicit; and (3) intervention intensity and complexity were considered important factors, as upper limb recovery, particularly in chronic patients, may require higher intensity, longer duration, or more frequent sessions than those typically provided in the included studies. Additionally, the inclusion of creative and functionally relevant VR tasks was deemed crucial (Amin et al., 2024). In future research, more precise definitions of patient subgroups (*e.g.*, by stroke severity or cognitive status) should be adopted, and standardized VR protocols should be employed to clarify differential effects.

### Daily function

VR was demonstrated to have a statistically significant positive effect on improving daily function in stroke patients, corroborating findings by Laver et al. (2017). Although the improvement was small, it may have clinical relevance as it can enhance independence and subjective well-being (Allen et al., 2002) and may reduce societal burden. Task-oriented practice was enabled by VR in simulated environments, thereby boosting self-care abilities (Wang et al., 2024). Although no significant moderating effects were observed, subgroup analyses indicated that training cycles of 5–8 weeks (Zhang, Wong & Qin, 2023) and session durations of 41–80 min were associated with significant within-group improvements. Additionally, immersive VR was found to provide a better treatment experience (Mekbib et al., 2020). However, patient type (*e.g.*, stroke severity, chronicity) and broad intervention categories were not found to have significant moderating effects. This lack of moderation was likely attributed to heterogeneity within these groups across studies. Future research is recommended to define patient subgroups more precisely (*e.g.*, by stroke severity or cognitive status) and to employ standardized VR protocols to elucidate differential effects.

### Balance

This meta-analysis demonstrated that VR interventions significantly improved balance in stroke patients, with a small but robust effect (Cohen’s d = 0.31, *P* < 0.01). Both fixed- and random-effects models yielded consistent results, suggesting that the effect was stable across studies. This finding aligns with Shen et al. (2023), who also reported significant postural and stability improvements following VR-based balance training. Several mechanisms may explain these improvements. (1) Enhanced sensory–motor integration: VR provides real-time visual and proprioceptive feedback, enabling patients to correct postural errors and improve weight-shifting control. (2) Increased engagement and task repetition: Interactive and gamified environments promote higher motivation and adherence, facilitating motor relearning. (3) Gradual progression of task complexity: Even though most included studies used relatively simple tasks (*e.g.*, Wii Fit balance games), the repeated and structured nature of training contributed to improvements in postural stability. Furthermore, the moderate heterogeneity (I^2^ = 29%) suggests that VR balance interventions were effective across diverse patient populations and program designs. These findings support the integration of VR into standard post-stroke balance rehabilitation programs to enhance recovery outcomes. Future research should focus on dynamic, ecologically valid tasks (*e.g.*, obstacle negotiation, dual-task balance challenges) and the use of more sensitive, instrumented balance measures to capture subtle improvements in postural control.

### Limitations of this study

This study has several limitations. First, methodological limitations in the primary studies, notably the inability to blind participants and high attrition rates, increased the risk of performance and attrition bias, particularly affecting non-significant outcomes, including upper limb function and balance. Inadequate allocation concealment and incomplete outcome reporting in some studies further compromised internal validity, potentially masking actual effects or introducing bias, especially for upper limb and balance results. Therefore, interpretation of these non-significant findings should be approached with caution.

Second, substantial clinical and methodological heterogeneity existed across studies, including variations in patient characteristics, training parameters, conventional therapies, VR systems, and outcome measures. Although subgroup analyses were conducted, limited data within specific subgroups may have reduced the precision of effect estimates. Nevertheless, sensitivity analyses supported the overall robustness of the results.

Third, language bias cannot be excluded because only studies published in English and Chinese were included. The omission of relevant studies published in other languages may have resulted from this restriction.

Fourth, the assessment of the sustainability of VR benefits was limited by the absence of long-term follow-up in the majority of trials.

Finally, the absence of standardized intervention protocols across studies hindered direct comparability and replication. Despite these limitations, valuable evidence supporting the effectiveness of VR in improving lower and upper limb motor function, daily function, and balance in stroke rehabilitation was provided by this meta-analysis.

## Conclusion

This meta-analysis of 27 RCTs demonstrated that significant improvements in lower limb motor function (Cohen’s d = 0.41, *P* < 0.001), upper limb motor function (Cohen’s d = 0.25, *P* = 0.03), daily function (Cohen’s d = 0.24, *P* = 0.01), and balance (Cohen’s d = 0.31, *P* < 0.001) in stroke patients were achieved through VR technology, supporting its integration into routine rehabilitation protocols. To optimize clinical outcomes, the following implementation strategies were recommended: (1) immersive VR interventions lasting 5–8 weeks, delivered three to five sessions per week at 40–60 min per session, targeting lower limb motor recovery and functional independence; (2) the use of haptic glove systems (*e.g.*, NeuroGlove) paired with task-specific games to address upper limb motor function limitations; and (3) the adoption of dynamic VR environments with perturbation to more effectively address balance impairments. Future research is needed to validate the long-term sustainability of treatment effects (>6 months), assess the cost-effectiveness of VR interventions across diverse healthcare settings, and develop standardized protocols for integrating emerging technologies.

##  Supplemental Information

10.7717/peerj.20402/supp-1 Supplemental Information 1 Basic features of the included studies

10.7717/peerj.20402/supp-2 Supplemental Information 2 ROB 2.0 Domain Assessments for Included Studies

10.7717/peerj.20402/supp-3 Supplemental Information 3 PRISMA checklist

10.7717/peerj.20402/supp-4 Supplemental Information 4 Audience

10.7717/peerj.20402/supp-5 Supplemental Information 5 Raw data

## Additional Informations and Declarations

### Data Deposition

The following information was supplied regarding data availability: The raw measurements are available in the Supplementary Files.
