# Supplementary material for: Virtual reality technology for upper and lower limb motor function, daily function, and balance in stroke patients: a meta-analysis of randomized controlled trials
Source: PeerJ. 2025 Dec 3;13:e20402. doi: 10.7717/peerj.20402 (PMC12681232; doi:10.7717/peerj.20402)
Supplement: Supplemental Information 1 [file peerj-13-20402-s001.docx]

Supplementary Table S1 Basic features of the included studies

| Author | Simple size(T/C) | Age | Intervention cycle, frequency, and duration | Indicators | Measurement tools |
| --- | --- | --- | --- | --- | --- |
| da Silva Ribeiro et al. (2015) | 30(15/15) | 18-60 | 8 weeks, 2 times/week, 60 min/time | motor function、balance, daily function | Fugl-Meyer, blance score, SF-36 |
| Ögün et al. (2019) | 65(33/32) | 61.48 ± 10.92 | 6 weeks, 3 times/week, 60 min/time | motor function, daily function | Fugl-Meyer, FIM |
| Bower et al. (2015) | 40(8/8) | mean=63.1 | 4 weeks, 8 times, 40 min/time | motor function, balance, daily function | Motor Assessment Scale, 6mWT, FIM |
| Chen et al. (2022) | 40(18/18) | 40-80 | 2 weeks, 5 times/week, 60 min/time | motor function | Fugl-Meyer |
| Ahmad et al. (2019) | 36(18/18） | mean=57 | 8 weeks, 1 time/week, 30 min/time | motor function, daily function | Fugl-Meyer , IADL |
| Choi & Cho (2024) | 46(23/23) | 55.69 ± 9.74 | 8 weeks,5 times/week, 40 min/time | motor function, daily function | Fugl-Meyer, MAL, MFT |
| Miclaus et al. (2021) | 59(31/28) |  | 10days,70 min/day | motor function, balance, daily function | Fugl-Meyer, TUG, FIM |
| Llorens et al. (2021) | 29(15/14) | 57.6 ± 6.9 | 3-5 times/week, 60 min/time | motor function | Fugl-Meyer |
| Adams et al. (2023) | 18(9/9) | 45-73 | 8 weeks,4 times/week, 45 min/time | motor function、daily function | Fugl-Meyer, MAL |
| Huang et al. (2022) | 30(15/15) | 20-75 | 16times,2-3 times/week,60 min/time | motor function | Fugl-Meyer |
| Park et al. (2019) | 25(12/13) | 53.5 ± 13.0 | 4 weeks,5 times/week, 60 min/time | motor function, daily function | Fugl-Meyer, MBI |
| Norouzi-Gheidari et al. (2019) | 18(9/9) | 42.2 ± 9.5 | 4 weeks,2 times/week, 44 min/time | motor function, daily function | Fugl-Meyer, MAL |
| Jo et al. (2024) | 45(15/15/15) | 51.73±13.63 | 4 weeks,3 times/week, 30 min/time | motor function | Fugl-Meyer |
| Sip et al. (2023) | 20(10/10) | 40-64 | 3 weeks,6 times/week, 30 min/time | motor function, daily function | Fugl-Meyer, SF-36 |
| Aşkın et al. (2018) | 40(18/20) | 53.27 ± 11.19 | 4 weeks,5 times/week, 60 min/time | motor function | Fugl-Meyer |
| Chen et al. (2021) | 23(12/11) | 30-70 | 3 weeks,5 times/week, 60 min/time | motor function, daily function | Fugl-Meyer, MAL |
| Lee, Shin & Song (2016) | 10(5/5) | 65.2 ± 5.0 | 4 weeks,5 times/week, 60 min/time | motor function, balance | Fugl-Meyer, BBS |
| Yatar & Yildirim (2015) | 30(15/15) | mean=62.8 | 4 weeks,3 times/week, 60 min/time | balance, daily function | BBS, FAI |
| Huh et al. (2015) | 40(23/17) | 61.51±0.92 | 2 weeks,5 times/week, 30 min/time | balance, daily function | BBS, MBI |
| Lee et al. (2012) | 40(20/20) | 53.75±11.29 | 4 weeks,5 times/week, 20 min/time | motor function, balance, daily function | FAC, BBS, MBI |
| Kim et al. (2018) | 30(12/11) | 56.7±17.8 | 2 weeks,5 times/week, 30 min/time | motor function, daily function | Fugl-Meyer, MBI |
| Song & Park (2015) | 40(20/20) | 51.37±40.6 | 8 weeks,5 times/week, 30 min/time | balance | TUG |
| In & Song (2016) | 25(13/12) | 57.31±10.53 | 4 weeks,5 times/week, 30 min/time | balance | BBS |
| Wan et al., 2025 | 30(14/16) | 18-75 | 4 weeks,5 times/week, 20 min/time | motor function, balance | Fugl-Meyer, BBS |
| Butcher et al., 2025 | 24(16/8) | SD=66.5 | 7 weeks,3 times/week, 30 min/time | motor function, daily function | Fugl-Meyer, MAL |
| Han et al., 2025 | 30(15/15) | 58.7±11.1 | 6 weeks,2 times/week, 30 min/time | balance | BBS |
| Ase et al., 2025 | 14(7/7) | 58.0± 6.4 | 4 weeks,5 times/week, 30 min/time | motor function, daily function | Fugl-Meyer, MAL |

Note: FMA-UE: Fugl-Meyer Assessment—Upper Extremity; FMA-LE: Fugl-Meyer Assessment—Lower Extremity; MAS: Motor Assessment Scale; FAC: Functional Ambulation Categories; FIM: Functional Independence Measure; IADL: Instrumental Activities of Daily Living; MFT: Manual Function Test; MMT: Manual Muscle Test; MAL: Motor Activity Log; MBI: Modified Barthel Index; SF-36: 36-Item Short Form Health Survey; FAI: Frenchay Activity Index; 6MWT: 6-Minute Walk Test; TUG: Timed Up and Go; BBS: Berg Balance Scale.
