# Supplementary material for: Virtual reality technology for upper and lower limb motor function, daily function, and balance in stroke patients: a meta-analysis of randomized controlled trials
Source: PeerJ. 2025 Dec 3;13:e20402. doi: 10.7717/peerj.20402 (PMC12681232; doi:10.7717/peerj.20402)
Supplement: Supplemental Information 2 [file peerj-13-20402-s002.docx]

Supplementary Table S2 ROB 2.0 Domain Assessments for Included Studies

| Study | Randomization process | Deviation from planned intervention measures | Result data missing | Bias in result measurement | Reporting bias |
| --- | --- | --- | --- | --- | --- |
| da Silva Ribeiro et al. (2015) | Low risk of bias (using computer-generated random numbers to allocate patients, hidden allocation plan, clear process description) | High risk of bias (some patients may not be able to complete all treatments due to personal reasons, but the specific number of dropouts and reasons for each group have not been reported in detail) | Uncertain (unclear whether there is missing data in the report and how it will be handled) | Low risk of bias (assessment tools are objective and reliable, and evaluators are unaware of patient grouping) | Low risk of bias (fully reported all relevant results) |
| Ögün et al. (2019) | Low risk of bias (using stratified randomization to ensure similar baseline characteristics between the two groups) | High risk of bias (some patients withdrew from the study prematurely due to pain or fatigue, but the number of dropouts in each group and their impact on the analysis were not specified in detail) | Uncertain (insufficient reporting of missing data and handling methods) | Low risk of bias (standardized assessment tools widely used in stroke rehabilitation research) | Low risk of bias (research results consistent with pre-set analysis plan) |
| Bower et al. (2015) | Low risk of bias (allocation of participants using computer-assisted randomization method) | High risk of bias (some patients may not be able to complete all game tasks due to personal preferences or physical conditions, but specific withdrawal situations are not specified) | Uncertain (no clear indication of whether there is data missing and how to handle it) | Low risk of bias (objective and standardized assessment tools) | Low risk of bias (comprehensive reporting of all preset assessment indicators) |
| Chen et al. (2022) | Low risk of bias (using computer software to assist in random allocation of patients to two groups) | High risk of bias (some patients may not be able to complete all interventions due to physical condition or other reasons, but the details of withdrawal are not specified) | Uncertain (insufficient reporting of missing data and handling methods) | Low risk of bias (assessment tools are objective and reliable, and evaluators are unaware of grouping) | Low risk of bias (research results consistent with pre-set analysis plan) |
| Ahmad et al. (2019) | Low risk of bias (simple randomization method is used to allocate participants, and the grouping process is clearly described) | High risk of bias (some patients may not be able to complete all interventions due to personal reasons, but the details of withdrawal are not specified) | Uncertain (no clear report on missing data and how to handle it) | Low risk of bias (standardized assessment tools and applicable to stroke rehabilitation assessment) | Low risk of bias (comprehensive report of all preset evaluation indicators) |
| Choi & Cho (2024) | Moderate risk of bias (grouping using computerized randomization program, but specific details of randomization not specified) | Moderate risk of bias (detailed description of intervention measures, but no mention of blinding) | Low risk of bias (no reported missing data) | Moderate risk of bias (without mentioning whether the evaluator is aware of the grouping situation) | Low risk of bias (research plan and report results are basically consistent) |
| Miclaus et al. (2021) | Moderate risk of bias (grouping using simple randomization method, but the description of randomization method is relatively brief) | Moderate risk of bias (detailed description of two intervention measures, but no mention of blinding) | Moderate risk of bias (5 participants withdrew, and the handling of missing data was not explained in detail) | Moderate risk of bias (without mentioning whether the evaluator is aware of the grouping) | Low risk of bias (no selective reporting issues found) |
| Llorens et al. (2021) | Moderate risk of bias (participants were randomly assigned, but specific details of randomization were not specified) | Moderate risk of bias (detailed description of intervention process, but blinding cannot be implemented) | Moderate risk of bias (some participants withdrew, and the handling and impact of missing data were not fully explained) | Moderate risk of bias (equipment operators and data collectors may be aware of grouping) | Low risk of bias (no selective reporting issues found) |
| Adams et al. (2023) | Moderate risk of bias (using a replacement block randomization scheme, but not detailing the randomization process) | Moderate risk of bias (detailed intervention and control measures, but no mention of blinding) | Moderate risk of bias (with participant withdrawal and lack of detailed explanation on the handling of missing data) | Moderate risk of bias (without mentioning whether the evaluator is aware of the grouping) | Low risk of bias (no selective reporting issues found) |
| Huang et al. (2022) | Moderate risk of bias (using online randomization table to randomly group, but insufficient description of randomization details) | Moderate risk of bias (detailed intervention measures but did not mention blinding of participants and treatment personnel) | Low risk of bias (no reported missing data) | Low risk of bias (detailed description of multiple outcome measurement indicators and evaluation methods, with blinded evaluators) | Low risk of bias (no selective reporting issues found) |
| Park et al. (2019) | Moderate risk of bias (computer-generated randomization sequence, but brief description of randomization details) | Moderate risk of bias (detailed intervention and control measures, but did not mention blinding of participants and treatment personnel) | Moderate risk of bias (1 participant withdrew without detailed explanation on the handling of missing data) | Low risk of bias (with clear evaluation criteria and evaluators, and evaluators being single blind) | Low risk of bias (no selective reporting issues found) |
| Norouzi-Gheidari et al. (2019) | Moderate risk of bias (the random sequence was generated by the recruiter through a "sealed envelope", but the method of generating the random sequence was not clearly described, and there was a significant difference in baseline age between groups) | Low risk of bias (unreported treatment adherence bias) | Moderate risk of bias (5 out of 23 participants in the intervention group withdrew, but intention to treat analysis was used) | Low risk of bias (evaluator blinding implemented well, using standardized tools) | Low risk of bias (all predetermined outcomes reported) |
| Jo et al. (2024) | Low risk of bias (clearly using computer-generated random sequences, allocation ratio 1:1:1, balanced baseline features) | Moderate risk of bias (the intervention group used VR devices and could not blind participants, but the control group used TMT to reduce performance bias) | Low risk of bias (no dropout cases, all 45 patients completed the study) | Low risk of bias (evaluator blinded, using validated scales) | Low risk of bias (with complete reporting of both primary and secondary outcomes) |
| Sip et al. (2023) | Moderate risk of bias (only mentioning "random allocation", without describing methods for generating or hiding random sequences, and with significant differences in baseline FMA-UE scores between groups) | Moderate risk of bias (no mention of blinding participants/therapists) | Low risk of bias (no dropout cases, all 20 patients completed the intervention) | Moderate risk of bias (unclear whether the evaluator was blinded, and subjective outcomes are susceptible to measurement bias) | Low risk of bias (all predetermined outcomes reported) |
| Aşkın et al. (2018) | Moderate risk of bias (grouping based on website generated random sequences, but without detailed instructions on how to execute random sequences and allocate hidden situations) | Low risk of bias (double-blind for patients and evaluators, with stimulation conducted by trained researchers and different personnel responsible for stimulation and evaluation) | Moderate risk of bias (1 patient withdrew, but the impact of withdrawal on the results was not explained in detail) | Low risk of bias (outcome measurement indicators are clear, evaluators are trained and unaware of grouping) | Low risk of bias (the article provides a detailed description of the study design, methods, and results) |
| Chen et al. (2021) | Moderate risk of bias (grouping using computer-generated random numbers, but without mentioning key information such as allocation concealment) | Moderate risk of bias (no mention of blinding) | Moderate risk of bias (with some patients withdrawing and a small sample size) | Moderate risk of bias (specific details of measurement and evaluators not specified) | Moderate risk of bias (no evidence of selective reporting was found, but due to the exploratory nature of the study, there may be unreported results) |
| Lee, Shin & Song (2016) | Moderate risk of bias (using simple randomization method, without mentioning allocation concealment) | Moderate risk of bias (not mentioned whether blinding was used) | Moderate risk of bias (although patients withdrew from the study and relevant statistical analysis was conducted, missing data may still affect the reliability of the results) | Moderate risk of bias (without mentioning whether the assessors were aware of the grouping) | Moderate risk of bias (the article presents the primary outcome, but there may be unreported secondary outcomes) |
| Yatar & Yildirim (2015) | Moderate risk of bias (randomized grouping by coin toss, no mention of allocation concealment) | Moderate risk of bias (not mentioned whether blinding was used) | Moderate risk of bias (some patients did not complete the study and the sample size was small) | Moderate risk of bias (without detailed explanation of the reliability of the measurement and the situation of the evaluators) | Moderate risk of bias (no evidence of selective reporting was found, but due to limitations in the study, there may be unreported relevant results) |
| Huh et al. (2015) | Moderate risk of bias (specific randomization methods and allocation concealment not mentioned) | Moderate risk of bias (patients and therapists not blinded) | Low risk of bias (no patients lost to follow-up, all participants completed the training plan) | Moderate risk of bias (without detailed description of evaluators) | Moderate risk of bias (the article presents the primary outcome, but there may be unreported secondary outcomes) |
| Lee et al. (2012) | Low risk of bias (generated by computer-generated random sequences and concealed allocation using opaque envelopes, operated by researchers who are not involved in patient selection, intervention, and evaluation) | Low risk of bias (using a double-blind design, both patients and assessors are unaware of grouping) | Moderate risk of bias (some patients withdrew from the study, although intention analysis was conducted, missing data may still affect the results) | Low risk of bias (using multiple standardized assessment tools, and the evaluator is unaware of the grouping) | Low risk of bias (detailed report of research results) |
| Kim et al. (2018) | Moderate risk of bias (no mention of randomization methods and allocation concealment) | Moderate risk of bias (not mentioned whether blinding was used) | Low risk of bias (no mention of patient loss to follow-up or missing data, assuming complete data) | Moderate risk of bias (without specifying whether evaluators are aware of the grouping) | Moderate risk of bias (presenting the main outcome, but there may be unreported related outcomes) |
| Song & Park (2015) | Moderate risk of bias (using random allocation software, but not mentioning allocation concealment) | Moderate risk of bias (no mention of blinding) | Moderate risk of bias (5 patients withdrew, sample size is small) | Low risk of bias (using multiple recognized assessment tools, trained evaluators, and unaware of grouping) | Low risk of bias (detailed report of all results) |
| In & Song (2016) | Moderate risk of bias (randomized grouping by coin toss, no mention of allocation concealment) | Moderate risk of bias (not mentioned whether blinding was used) | Moderate risk of bias (some patients did not complete the study and the sample size was small) | Moderate risk of bias (without detailed explanation of the reliability of the measurement and the situation of the evaluators) | Moderate risk of bias (no evidence of selective reporting was found, but due to limitations in the study, there may be unreported relevant results) |
| Wan et al. (2025) | Moderate risk of bias (Randomization method not specified in detail, allocation concealment unclear) | Moderate risk of bias (No mention of blinding of participants or therapists, performance bias possible) | Low risk of bias (No missing data reported, complete follow-up) | Moderate risk of bias (No mention of blinding of outcome assessors) | Low risk of bias (All preset outcomes reported according to methods section) |
| Butcher et al. (2025) | Moderate risk of bias (Randomization method not clearly described, allocation concealment procedures unclear) | Moderate risk of bias (No blinding of participants or personnel mentioned) | Moderate risk of bias (Some participants withdrew, but handling of missing data not fully explained) | Moderate risk of bias (Blinding of outcome assessors not mentioned) | Low risk of bias (All predetermined outcomes appear to be reported) |
| Ase et al. (2025) | Moderate risk of bias (Randomization method briefly mentioned but details insufficient) | Moderate risk of bias (Participants and therapists not blinded to intervention) | Low risk of bias (No missing data reported, all participants completed study) | Low risk of bias (Used standardized assessment tools with established reliability) | Low risk of bias (Comprehensive reporting of all outcomes) |
| Han et al. (2025) | Moderate risk of bias (Randomization procedure not adequately described) | Moderate risk of bias (No mention of blinding procedures for participants or therapists) | Low risk of bias (No dropouts or missing data reported) | Moderate risk of bias (Blinding of outcome assessors not specified) | Low risk of bias (All planned outcomes reported) |
