# Supplementary material for: Virtual reality technology for upper and lower limb motor function, daily function, and balance in stroke patients: a meta-analysis of randomized controlled trials
Source: PeerJ. 2025 Dec 3;13:e20402. doi: 10.7717/peerj.20402 (PMC12681232; doi:10.7717/peerj.20402)
Supplement: Supplemental Information 4 [file peerj-13-20402-s004.docx]

Stroke is a common nervous system disease, which often leads to motor dysfunction, seriously worsening the quality of life of patients and increasing economic burden. As an innovative rehabilitation treatment technology, virtual reality technology has been used in the rehabilitation training of stroke patients. This study systematically evaluated the influence of virtual reality technology on motor function, daily function and balance of stroke patients. Through this study, we hope to shed lights on the intervention of stroke patients, promote the research progress in related fields, and provide practical solutions to the rehabilitation and social adaptation of stroke patients.
